# Sperm Cryopreservation before Testicular Cancer Treatment and Its Subsequent Utilization for the Treatment of Infertility

**DOI:** 10.1155/2014/575978

**Published:** 2014-01-22

**Authors:** Jana Žáková, Eva Lousová, Pavel Ventruba, Igor Crha, Hana Pochopová, Jaroslava Vinklárková, Eva Tesařová, Mohamed Nussir

**Affiliations:** ^1^Department of Gynaecology and Obstetrics, Faculty of Medicine, Masaryk University and Faculty Hospital Brno, Obilní trh 11, 602 00 Brno, Czech Republic; ^2^Transfusion and Tissue Department, Faculty Hospital Brno, Jihlavská 20, 625 00 Brno, Czech Republic; ^3^Department of Urology, Faculty of Medicine, Masaryk University and Faculty Hospital Brno, Jihlavská 20, 625 00 Brno, Czech Republic

## Abstract

*Aims.* In this study we report our results with storage of cryopreserved semen intended for preservation and subsequent infertility treatment in men with testicular cancer during the last 18 years. *Methods.* Cryopreserved semen of 523 men with testicular cancer was collected between October 1995 and the end of December 2012. Semen of 34 men (6.5%) was used for fertilization of their partners. They underwent 57 treatment cycles with cryopreserved, fresh, and/or donor sperm. *Results.* A total of 557 men have decided to freeze their semen before cancer treatment. Azoospermia was diagnosed in 34 men (6.1%), and semen was cryopreserved in 532 patients. Seminoma was diagnosed in 283 men (54.1%) and nonseminomatous germ cell tumors in 240 men (45.9%). 34 patients who returned for infertility treatment underwent 46 treatment cycles with cryopreserved sperm. Totally 16 pregnancies were achieved, that is, 34.8% pregnancy rate. *Conclusion.* The testicular cancer survivors have a good chance of fathering a child by using sperm cryopreserved prior to the oncology treatment, even when it contains only limited number of spermatozoa.

## 1. Introduction

According to the data obtained from the National Oncologic Registry of ÚZIS ČR the incidence of testicular cancer is increasing worldwide. Czech Republic is on the 7th place among 182 countries of the world in the incidence 9.7 testicular cancer/100,000 males. Testicular cancer is the most common malignancy in the age group from 20 to 40 years, where the incidence in the age group between 25 and 29 years is 19.4% and from 30 to 34 years 19.6% [1]. Testicular cancer patients have a very good prognosis for survival since young patients dominate. Therefore it is important to consider the consequences of the treatment that may affect their fertility, just before initiation of cancer treatment. Germ cell tumours of an adults testis are generally classified into two main groups on the basis of histological, serological, and clinical data: (i) seminoma and (ii) nonseminoma germ cell tumours (NSGCT), which include embryonal carcinoma, yolk sac tumours, polyembryoma, choriocarcinoma, and teratomas [2].

Fertility after the cancer treatment is variable and usually depends on the chemotherapy and radiotherapy used and/or size of the radiation field, dose, and dose intensity. Method of administration, age, and pretreatment fertility of the patient play also important role [3, 4]. The consequent measurable effects of chemotherapy or radiotherapy include compromised number of spermatozoa, their motility, morphology, and/or DNA integrity.

The basic method used to preserve reproductive potential of the survivors of cancer treatment is cryopreservation of the sperm before gonadotoxic therapy. The advances in the field of assisted reproduction techniques and sperm banking allowes semen storage even in men with low sperm quality. Sperm could be used for intrauterine insemination, when quality is fair by *in vitro* fertilization or by the Intracytoplasmic Sperm Injection (ICSI), when concentration and/or motility is low.

Large prospective or even retrospective studies comparing the outcome of assisted reproduction with sperm from oncological patients are rare in the literature. Only a few studies analyzed the utilization of cryopreserved sperm by male cancer survivors. For instance, in retrospective review from 1992 [5] 43 out of 191 couples (22.5%) conceived after artificial insemination using husband's frozen-thawed semen and 10 out of 12 couples conceived after IVF. Also in other study IVF achieved higher success rate than insemination and ICSI was more successful than conventional IVF [6]. Large retrospective series reported on 64 patients which had received either intrauterine insemination (IUI) or conventional IVF and/or ICSI as described in 2001 [7]. In study of Magelssen et al. [8] only in 29 men out of 422, that is, 7%, their cryopreserved semen was used for ART.

Many case reports were reported with successful outcome: Hakim et al. [9] reported the achievement of pregnancies using assisted reproduction for male factor infertility after retroperitoneal lymph node dissection for testicular carcinoma. Chen et al. [10] reported pregnancy achieved using sperm administered by ICSI, from male with testicular cancer. Live birth with sperm cryopreserved and stored for 21 years prior to cancer treatment was reported in 2004 [11].

Here we report results from the Centre of Assisted Reproduction Centre at the Department of Gynaecology and Obstetrics, Faculty of Medicine, Masaryk University and the Faculty Hospital in Brno, which is the first Czech Centre of Assisted Reproduction, where the first Czech test tube baby was born in 1982. This Centre is also the first workplace, where the program of sperm collection, freezing, and long-term storage began in 1995 in collaboration with Urology Department Faculty Hospital in Brno, Masaryk Memorial Cancer Institute Brno, St. Anne's University Hospital Brno and Transfusion and Tissue Department of Faculty Hospital Brno.

In this report we reviewed our results with storage of cryopreserved semen for fertility preservation and subsequent infertility treatment in men with testicular cancer. We analyze the sperm counts and possible correlation between sperm pathology and cancer diagnosis and make an overview of the use of the frozen sperm for assisted reproduction during the last 18 years of sperm banking.

## 2. Materials and Methods

Young men with testicular cancer were referred (between October 1995 and the end of December 2012) from three Departments of Urology to our Assisted Reproduction Centre for sperm cryopreservation prior to the treatment of testicular cancer. All patients signed their informed consent to cryopreservation.

Specimens were produced by masturbation into a sterile container and allowed to liquidify for 30 min before analysis. Semen analysis was performed according to the WHO (World Health Organization) laboratory manual [12–14] using the Bürker or Makler counting chamber. Sperm freezing was carried out after dilution into a cryoprotectant medium Medi-Cult, respectively, Origio (Denmark), taking into account the number of spermatozoa and their motility. Semen was mixed with a cryoprotectant and divided in 1.0 mL volume cryotubes or 1.8 mL volume cryotubes Nunc (Denmark).

Cryopreservation technology and the procedures used in the storage of frozen sperm samples were aimed at minimizing the risks of potential transmission of infection. Prescreening was required and the patients were checked for Hepatitis B and C, HIV, and syphilis. Sperm samples were from 1995 to 2004 frozen in the programmable freezer Planer Kryo F10 (Sunbury-On-Thames, UK) using a standard cooling curve. From July 2004 samples were cryopreserved in nitrogen vapour only. We did not observe significant differences in motility or viability with the two methods of cryopreservation. Usually, sample collections from 1 to 3 were frozen before starting cancer treatment. The cryotubes were stored in liquid nitrogen at −196°C in MVE XC Dewar liquid nitrogen tank, Tailor-Wharton.

For assisted reproduction, frozen semen samples were first thawed at room temperature and allowed to recuperate for 20 min. Thawed semen samples were then washed twice with washing medium to eliminate seminal plasma. Motile spermatozoa were collected by a “swim-up” procedure. The final suspension was kept in the incubator in 37°C for 2 h before insemination. Such sperm was used either for intrauterine insemination or for oocyte fertilization by classic IVF or ICSI methods.

Statistics—the study group has been described using basic descriptive statistics, where categorical variables were characterized using the percentage representations of individual categories, while continuous variables (age, sperm concentration, and motility) were described using the mean, the median, the range of values (minimum and maximum), and standard deviations. Statistical testing was used to confirm the hypothesis of whether or not the results of sperm counts correlate with the patient's diagnosis. The differences among group of patients were tested using the Kruskal-Wallis test. The critical limit for the level of significance was set to *P* = 0.05.

## 3. Results and Discussion

**Figure 1:**
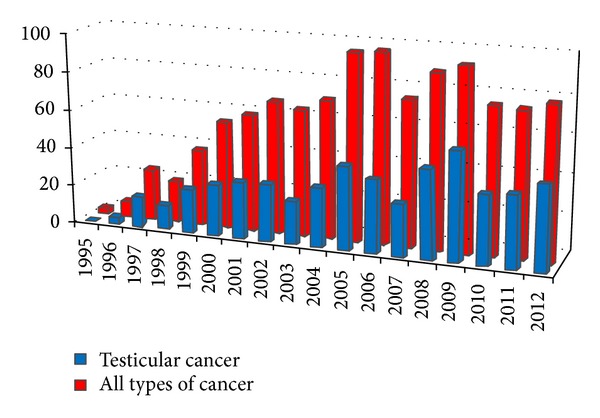
Number of men with cryopreserved semen by years.

Semen of 523 men with testicular cancer was cryopreserved from 1995 to the end of 2012, that is 47% from all types of cancer patients (1111 men) (Figure 1).

**Figure 2:**
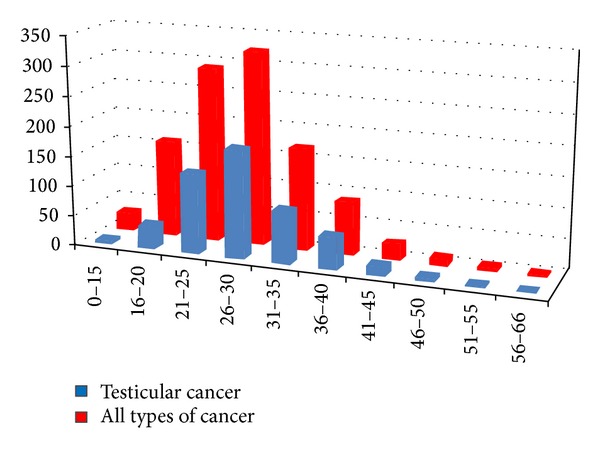
Number of men with cryopreserved semen by the age.

557 men decided to freeze their semen before testicular cancer treatment, but azoospermia was diagnosed in 34 men (6.1%), excluding these men from the possibility to cryopreserve and subsequently use of their samples for fertility treatment. Normospermia was diagnosed only in 31 men (5.6%). Oligoasthenoteratozoospermia was diagnosed, in 296 men (53.1%) and oligozoospermia or asthenozoospermia in 296 men (35.2%). The mean age in these men was 28.5 years, SD ± 6.6, range 13–64, median 28. Most often was semen cryopreserved in the age from 26 to 35 years (Figure 2). Seminoma was diagnosed in 283 men (54.1%), nonseminomatous germ cell tumors (NSGCT) in 240 men (45.9%). Until now, only 14 patients (2.5%) died.

Sperm concentration was determined 16.8 ± 19.8 mil/mL in seminoma group and 19.7 ± 19.9 mil/mL in NSGCT, which is considered be a nonsignificant difference. Progressive sperm motility (10.6 ± 13.5% in seminoma group and in NSGCT 12.9 ± 13.4%) was again considered to be a nonsignificant difference irrespective of tumor stage (Table 1). Statistical analysis did not reveal any significant difference of sperm count in relation to the histologic diagnosis of the cancer type.

Until now, 15 patients (2.9%) died. 34 men (6.5%) with their partners returned for infertility treatment and they underwent 57 treatment cycles: 46 with cryopreserved sperm, 3 with fresh sperm and in 8 cases after repeated unsuccessful attempt with partner's cryopreserved/thawed sperm, a donor sperm was used. These patients have been subjected to standard chemotherapy or radiotherapy treatments and they usually did not achieve natural conception.

The interval between cryopreservation and infertility treatment was in the range 7–70 months (mean 22.2 ± 14.7, median 18 months).

A small group of 6 IUI cycles resulted in 3 pregnancies (50.0% pregnancy rate) with one delivery. 38 ICSI cycles resulted in 13 pregnancies (34.2% pregnancy rate) with five deliveries and 2 IVF with donated eggs remained without pregnancy. At three cycles IVF + fresh sperm was obtained with 33.3% pregnancy rate with one delivery. Eight cycles with donor sperm remained without pregnancy, the deep problems manifested at female side. These results are summarised in Table 2.

Although male cancer survivors can become parents through options such as adoption or sperm donation, most prefer to have a biological offspring, even if they have concerns about birth defects that could be caused if the man had cancer before conception or anxiety about their own longevity or their child's lifetime cancer risk [15, 16]. Advances in diagnostics and management of this cancer and progress in the treatment contribute to the significant improvement in the survival rate of this very common malignancy in young men [17]. At our workplace azoospermia was found in 6.1% of males referred for sperm cryopreservation as compared to 17.3% [18] and to only 3.3% [7]. The reduction in sperm concentration and progressive motility were detected in both seminoma and NSGCT group similarly to other studies [19, 20]. Currently, the aetiology of impaired spermatogenesis in testicular cancer patients is not fully understood. Serious damage to the DNA of sperm due to the malignancy was confirmed [21]. However some other work did not report significant differences in mean sperm DNA fragmentation index within cancer subgroups or when comparing testicular and nontesticular cancers and the measured parameters in cancer subgroups did not differ from healthy men [19]. The correlation between sperm pathology and testicular tumours is also known [22]. The impaired quality of sperm production is probably associated with disturbed differentiation of the testicle during the embryonic development of the gonad according to Testicular Dysgenesis Syndrome (TDS) hypothesis [23]. TDS syndrome is manifested by the increased incidence of developmental defects of the genital (cryptorchism, hypospadias), spermatogenesis disorders and testicular carcinomas. Testicular dysgenesis is caused by alteration in the development of the testicle, being determined by the factors affecting endocrine regulation (“endocrine disruptors”). As spermatogenesis disorders correlate well with testicular carcinoma, close urological examination of men with severe sperm abnormalities is of importance. When analysing impaired spermatogenesis in relation to the type of malignancy, we did not find a significant difference (in contrast to Agarwal et al. [24]).

After the completion of gonadotoxic therapy, the quality of sperm was significantly impaired. The effect of radiotherapy on male fertility is clearly dose dependent. The application of radiation to tests greater than 6 Gray will result in irreversible azoospermia. At levels of 3.5 Gray, sterility does occur. The loss of fertility is usually reversible although commonly such recovery will take 18 to 24 months [25]. Sperm cryopreservation performed prior to cancer therapy is therefore a prerequisite for the successful treatment of subsequent infertility. The main requirement of this programme is to establish a special cryobank to allow safe long-term storage of sperm samples. The operation of such a cryobank requires an exact database of patients and meticulous maintenance of its own records. Another important aspect is that the patients should be given clear and relevant information on the possibilities and conditions of sperm cryopreservation and its use in the future.

The fulfilment of this task requires close cooperation between the Department of Urology and Assisted Reproduction Centre capable of providing this kind of treatment. In the Faculty Hospital Brno, this interdisciplinary cooperation was effectively facilitated [26]. Thanks to a strong awareness among urology specialists and availability of sperm cryopreservation in our Centre, the number of patients referred for this procedure has quickly increased.

In our study only 6.5% of males have come for infertility treatment so far; this finding is similar to data reported in the literature, for example, 7.7% [7]. Pacey et al. reported the utilization rates of banked sperm as very low (<10%) and the majority of samples were kept for many years without being used [27]. By UK study, to date very few (4%) of their patients chosen to discard, use; or move to another centre [28].

The reasons are not only in the area of patient health, but also in the social area; that is, patients usually plan to start a family long after they have successfully completed therapy [29]. Most males from our group who came for infertility treatment had undergone successful treatment for testicular cancer and usually came 18 months after sperm cryopreservation. One study in men suggested that having banked sperm was a positive factor for coping emotionally with cancer, even if samples were never used [30]. The similar beneficial role of psychological support plays ovarian tissue cryopreservation in young women suffering from cancer [31, 32].

Intrauterine insemination was performed in our clinic much less frequently (13.0%) and ICSI was used in 82.6% of treatment cycles. Only 2.7% males referred for sperm cryopreservation died, which corresponds to the total survival rate in patients with the early stage of testicular seminoma, which exceeds 95% [33].

## 4. Conclusions

The testicular cancer survivors have a good chance of fathering a child by using sperm cryopreserved prior to the oncology treatment thanks to assisted reproduction methods. In the ICSI era, almost any cryopreserved semen sample, even when it contains only few sperm, could be used for subsequent infertility treatment. Semen preservation before the beginning of therapy should be proposed to all adult men and postpubertal boys. To date, no clinically proven methods are available to preserve fertility in prepubertal males. The hope is that one day science will provide a mechanism for immature germ cells from the testicular tissue of those patients to be used *in vivo* or *in vitro* to facilitate reproduction. Sperm banking program requires close cooperation between assisted reproduction centers and the cancer clinics.

## Figures and Tables

**Table 1:** The semen analysis before cryopreservation.

	Seminoma (*n* = 283)	NSGCT* (*n* = 240)	Statistic	Totally (*n* = 523)
Sperm concentration				
Mean	16.8	19.7	NS**	18.4
Range	0–122	0–110		0–122
SD	19.8	19.9		19.9
Progressive sperm motility (%)				
Mean	10.6	12.9	NS**	11.8
Range	0–60	0–60		0–60
SD	13.5	13.4		13.3

*NSGT: nonseminomatous germ cell tumors.

**NS: statistically nonsignificant.

**Table 2:** Methods provided with cryopreserved/thawed sperm and fresh sperm samples.

Method	Number of cycles	Number of pregnancies	Pregnancy rate (%)	Number of deliveries
IUI	6	3	50.0	1
ICSI	38	13	34.2	5
IVF + D.O.*	2	0	0.0	0
ICSI *+ *fresh	3	1	33.3	1
ICSI + AID**	8	0	0.0	0

*Donor oocyte.

**Donor sperm.
